# Determination of Graphene Oxide Adsorption Space by Lysozyme Uptake—Mechanistic Studies

**DOI:** 10.1021/acs.jpcb.1c08294

**Published:** 2022-01-25

**Authors:** Paulina Erwardt, Katarzyna Roszek, Marek Wiśniewski

**Affiliations:** † Faculty of Chemistry, Physicochemistry of Carbon Materials Research Group, Nicolaus Copernicus University in Toruń, Gagarina 7, 87-100 Toruń, Poland; ‡ Department of Biochemistry, Faculty of Biological and Veterinary Sciences, Nicolaus Copernicus University in Toruń, Lwowska 1, 87-100 Toruń, Poland

## Abstract

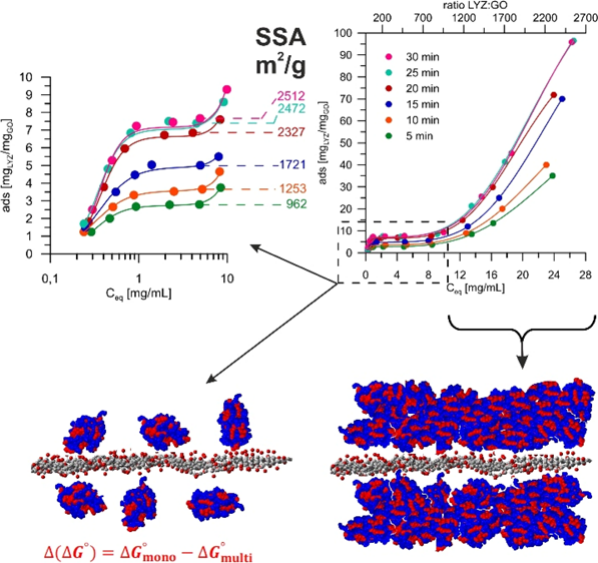

The interaction between graphene oxide (GO) and lysozyme (LYZ) in aqueous solution was investigated for GO specific surface area determination and for the thermodynamic description of the process. It was experimentally proved that LYZ is a much better adsorbate than the most common methylene blue, allowing the determination of genuine GO surface area. Our fluorescence spectroscopy results indicate that LYZ molecules interact with GO at high- and low-affinity sites depending on the surface coverage, reflecting the protein mono- and multilayer formation, respectively. The lack of the secondary structure changes confirms LYZ usability as a model adsorbate. The calculated values of thermodynamic parameters (Δ(Δ*H*^0^) = −195.0 kJ/mol and Δ(Δ*S*^0^) = −621.3 J/molK) indicate that the interactions are exothermic, enthalpy-driven. All the reported results reveal the physical nature of the LYZ–GO interaction at the studied concentration ratios.

## 1 Introduction

Graphene oxide (GO) is one of the most studied carbonaceous materials owing to its exceptional physicochemical and biological features. Thus, GO has been commonly referred to for biomedical applications, including but not limited to biosensing, drug delivery, and imaging, as well as for the control of stem cell differentiation. The fundamental interactions between the GO surface and biomolecules, active compounds, drugs, and cells strongly influence the biological pathways; however, the mechanism of such influence remains unknown.^1,2^ The drug and gene delivery approach relies on the successful adsorption of the plethora of molecules on the surface of GO structure, thus offering high potential as a delivery carrier.^1^ Additionally, graphene-based materials remain highly successful in the cell and tissue engineering field for the regeneration of various tissues. The most exciting findings include nerve, cartilage, skeletal muscle, cardiac tissue, and skin regeneration, as well as GO effects on the induced pluripotent stem cell cytophysiology.^3^ However, some challenging issues of GO application, such as long-term toxicity or biodegradability, have impeded its widespread access to clinics.

For each of the abovementioned applications, the GO specific surface area (SSA) determination is needed. However, the proper area seems to be extremely hard to study; thus, it is usually under- or overestimated. Unfortunately, GO origin from graphite determines how this critical parameter is usually investigated. Calculating the SSA from the low-temperature N_2_ adsorption isotherms is the fundamental method for carbons (hard and stable materials that do not change the structure during the drying process). However, in the case of soft and flexible materials such as GO, aggregation via “sticking” of the separate sheets (even during lyophilization) occurs, implying that the method is not suitable. Thus, it seems highly reasonable to perform the SSA determination in a natural environment, that is, in aqueous solution, excluding the desiccation stage. Nevertheless, in this case, particular care should also be taken to avoid aggregation of the separate GO sheets.

Another crucial factor is the adsorbate used. The literature data obtained for the most often used adsorbate - methylene blue (MB)–prove that, using this model molecule, the maximal SSA determined is lower than 50% of the theoretical value, 2630 m^2^/g^4^ (Table S1). The first conclusion is that MB is a comfortable but not a good adsorbate; the calculated SSA values are scattered drastically from 42 up to ca. 1200 m^2^/g. The other conclusion is hidden in the adsorbent (GO) preparation. By harnessing low-power ultrasound (US) as the dispersing method, better results can be obtained. Moreover, there is a competition between dispersion and deoxygenation - the low power (also short-time operation) disperses the GO sheets without reducing the surface functionalities. As the deoxygenation process triggers an increase in hydrophobicity, agglomeration via increase in π–π interactions and the consequent decrease in SSA are observed (Table S1).

In the present study, we aimed to prove the possibility of proper, genuine GO surface measurements using the small, rigid protein—lysozyme as the adsorbate. Applying the combination of adequate adsorbate molecules and the appropriate US power allowed for the simple experimental confirmation of the theoretical SSA value of ca. 2630 m^2^/g. Harnessing fluorescence spectroscopy, Fourier transform infrared (FTIR), and thermodynamic analyses disclosed the kinetics and nature of LYZ–GO interaction.

## 2 Materials and Methods

### 2.1 GO Preparation

GO was synthesized by a modified Hummers method^5,6^ and described previously.^7−9^ Briefly, 50 mL of concentrated H_2_SO_4_ was added to graphite flakes (0.175 g). KMnO_4_ (2.25 g) was slowly added to the suspension. The reaction mixture was kept at 25 °C and stirred for 24 h. Oxidation was stopped by adding 5 mL of 30% H_2_O_2_. After that, the mixture was centrifuged (at 8000*g* for 5 min). The remaining solid material was washed several times with 200 mL of 30% HCl and with 500 mL of water. After each wash, the mixture was centrifuged (at 15,000*g* for 20 min). The final resulting material was freeze-dried for 24 h. Before use, GO was diluted in deionized water and ultrasonicated for 60 min to obtain GO solution.

### 2.2 MB Adsorption

The GO solutions with 0.1 mg/mL concentration were exposed to sonication for 5, 10, 15, and 20 min. The same amount of GO (100 μL = 0.01 mg) was added to dark bottles containing MB solutions with different concentrations (0.0015–0.05 mg/mL). The mixtures were shaken at 120 rpm in a thermostated shaker for 24 h at 298 K. After reaching equilibrium, the solutions were centrifuged at 10,000 rpm for 15 min at 298 K. The concentration of the obtained supernatants was measured using a Jasco V-660 UV–vis spectrophotometer (Jasco Corporation, Tokyo, Japan) in a wavelength range of 450–800 nm.

### 2.3 LYZ Adsorption

The protein adsorption studies were performed similarly; the only differences were as follows: (i) additional sonication times of 25 and 30 min; (ii) maximal LYZ concentration of 30 mg/mL (in 0.9% NaCl); (iii) to reach equilibrium, the mixtures were shaken at 120 rpm in a thermostated shaker for 24 h at 277 K; and (iv) for quantitative measurements, the area of the 280 nm band was used.

### 2.4 Fluorimetric Measurements

The fluorescence spectra were measured with a fluorescence spectrometer RF-5001 PC (Shimadzu, Japan). The emission spectra were recorded in the range of 250–600 nm at an excitation wavelength of 295 nm. The fluorescence spectra were measured at the temperature range of 23.5–18.0 °C.

### 2.5 Attenuated Total Reflection FTIR Analysis

FTIR spectroscopy data were acquired by using a Vertex V70 (Bruker Optics) system, in attenuated total reflection (ATR) mode (single reflection using diamond crystal), in the frequency range 6000–15 cm^–1^.

## 3 Results and Discussion

**Figure 1:**
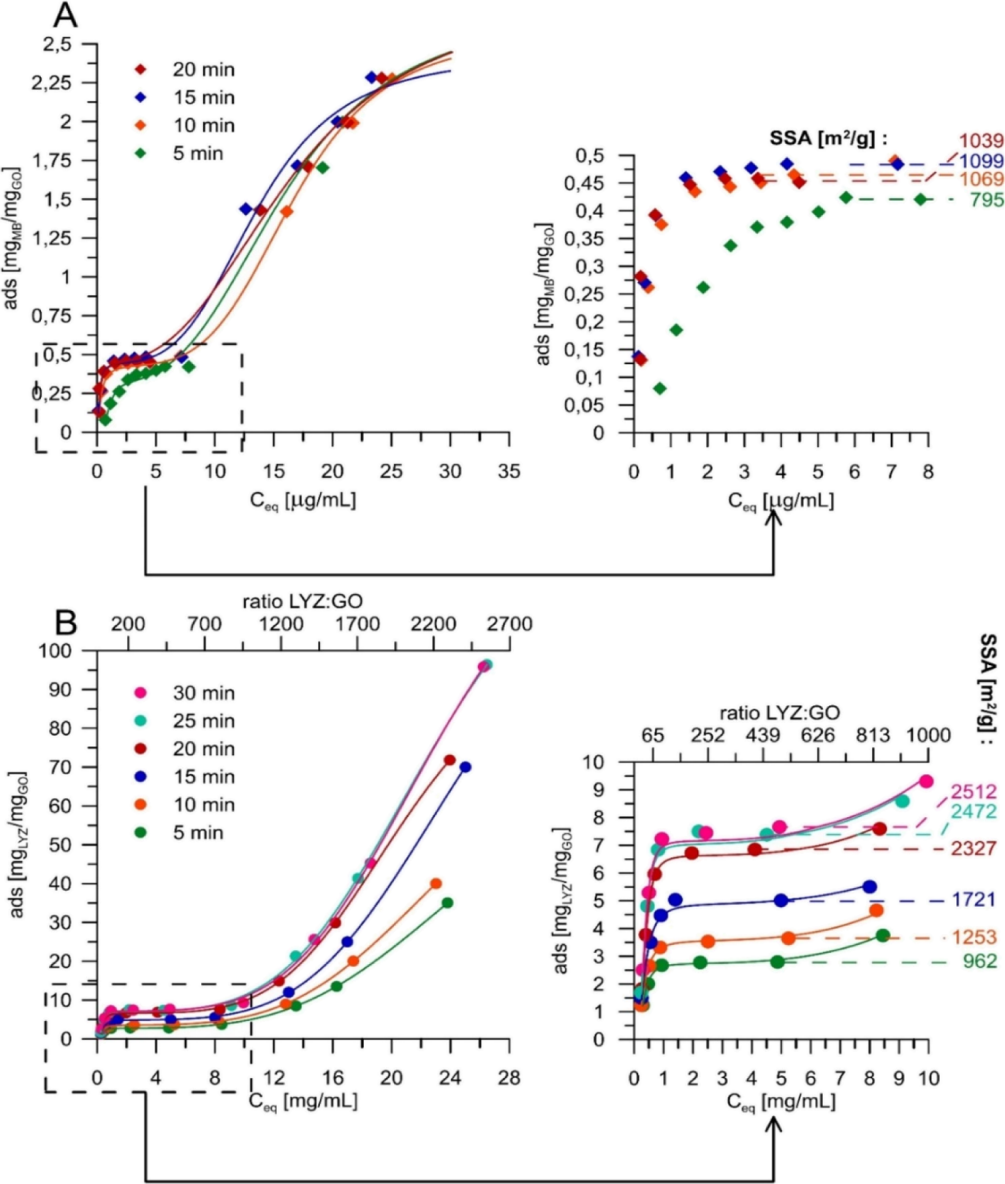
Influence of sonication time on (A) MB and (B) LYZ adsorption isotherms. Note that fits’ components are presented in Figure S1.

MB as a model molecule has been adsorbed on the GO surface in a wide concentration range. The obtained results are displayed in Figure 1A and Tables 1 and S1, which are typical and in agreement with other data. The SSA, depending on the sonication time, increases from ca. 800 to 1100 m^2^/g.

**Table 1:** Fitted Parameters of the Bimodal Langmuir–Freundlich Equation (SD Values are Shown in Parentheses)

adsorbate	time of sonication [min]	*Q*_m,1_ [g/g_GO_]	*K*_1_ [L/g]	*n*_1_	*Q*_m,2_ [g/g_GO_]	*K*_2_ [L/g]	*n*_2_	*R*^2^
MB	5	0.325 (0.113)	962.949 (44.67)	0.315 (0.427)	2.411 (0.804)	63.043 (1.355)	0.319 (0.035)	0.9876
	10	0.436 (0.034)	3126.465 (65.34)	0.646 (0.243)	2.137 (0.218)	59.786 (0.271)	0.238 (0.050)	0.9985
	15	0.448 (0.069)	4762.000 (221.20)	0.552 (0.460)	1.979 (0.335)	73.425 (0.795)	0.271 (0.089)	0.9897
	20	0.458 (0.050)	4997.317 (296.68)	0.691 (0.071)	2.345 (0.085)	60.971 (1.521)	0.355 (0.017)	0.9910
LYZ	5	2.745 (0.055)	3.172 (0.120)	0.3722 (0.050)	84.57 (2.68)	0.0370 (0.0018)	0.2649 (0.0084)	0.9999
	10	3.576 (0.069)	3.155 (0.139)	0.4310 (0.042)	87.65 (3.83)	0.0400 (0.0012)	0.2475 (0.0064)	0.9999
	15	4.910 (0.165)	2.778 (0.213)	0.4232 (0.068)	132.71 (9.63)	0.0397 (0.0018)	0.2291 (0.0104)	0.9999
	20	6.640 (0.227)	2.704 (0.149)	0.3092 (0.057)	101.05 (6.87)	0.0475 (0.0016)	0.2178 (0.0114)	0.9999
	25	7.052 (0.651)	2.876 (0.499)	0.3071 (0.148)	142.63 (11.26)	0.0428 (0.0033)	0.2425 (0.0253)	0.9996
	30	7.167 (0.702)	2.763 (0.459)	0.2838 (0.171)	143.90 (12.58)	0.0426 (0.0033)	0.2348 (0.0257)	0.9995

The very important but narrow (3–6 μg/mL) MB concentration range responsible for monolayer formation (Figures 1A and S1) can be easily omitted. Consequently, it leads to SSA calculations based on higher adsorbate concentrations, and the values obtained are highly overestimated (3000–6000 m^2^/g), resulting from the multilayer presence.^10−12^

Lysozyme (LYZ) is a small (14.4 kDa), rigid, and ellipsoid-shaped protein, with the adsorptive area depending on the “side-on”, “between”, or “end-on” orientation: 10.59, 7.49, and 7.07 nm^2^, respectively.^13^ Assuming the LYZ random adsorption on the GO surface, the average area value is 8.38 nm^2^.

Based on this, the GO SSA has been determined to be ca. 2600 m^2^/g at the theoretical level, confirming that the surface area was calculated for monolayer, but not for multilayered, adsorption (Figure 1B).

The bimodal Langmuir–Freundlich equation successfully fits all the experimental data (Figures S1 and S2). This simple model was presented as applicable for describing different adsorption data (see, e.g., Huang et al.^14^ Also, in our previous study,^7^ the model was harnessed to quantify catalase adsorption on different carbonaceous materials with reasonable accuracy.

The model is represented by the expansion of the equation proposed by Jeppu and Clement^15^ and Umpleby et al.^16^ to the bimodal form
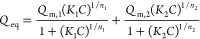
1where *Q*_eq_ is the amount of adsorbate adsorbed at equilibrium (g_LYZ_/g_GO_); *Q*_m_ is the maximum adsorbed capacity of the system (g_LYZ_/g_GO_); *C* is the protein concentration in solution at equilibrium (g/L); *K* is the affinity constant between the adsorbate and the adsorbent (L/g), and *n* is the index of heterogeneity.

The fitted values of *Q*_m,*x*_, constant *K*_*x*_, and the parameter *n*_*x*_ (*x* = 1,2) are summarized in Table 1. The value of *n* = 1 suggests noninteracting sites, while 0 < *n* < 1 the positive cooperativity, whereas for *n* > 1, negative cooperativity is expected during the adsorption process.

The left part of Table 1 describes the sites with higher GO surface-to-adsorbate affinity, typical for monolayer formation. Contrarily, the right side defines multilayer formation. It dominates at a higher *C*_eq_ range.

**Figure 2:**
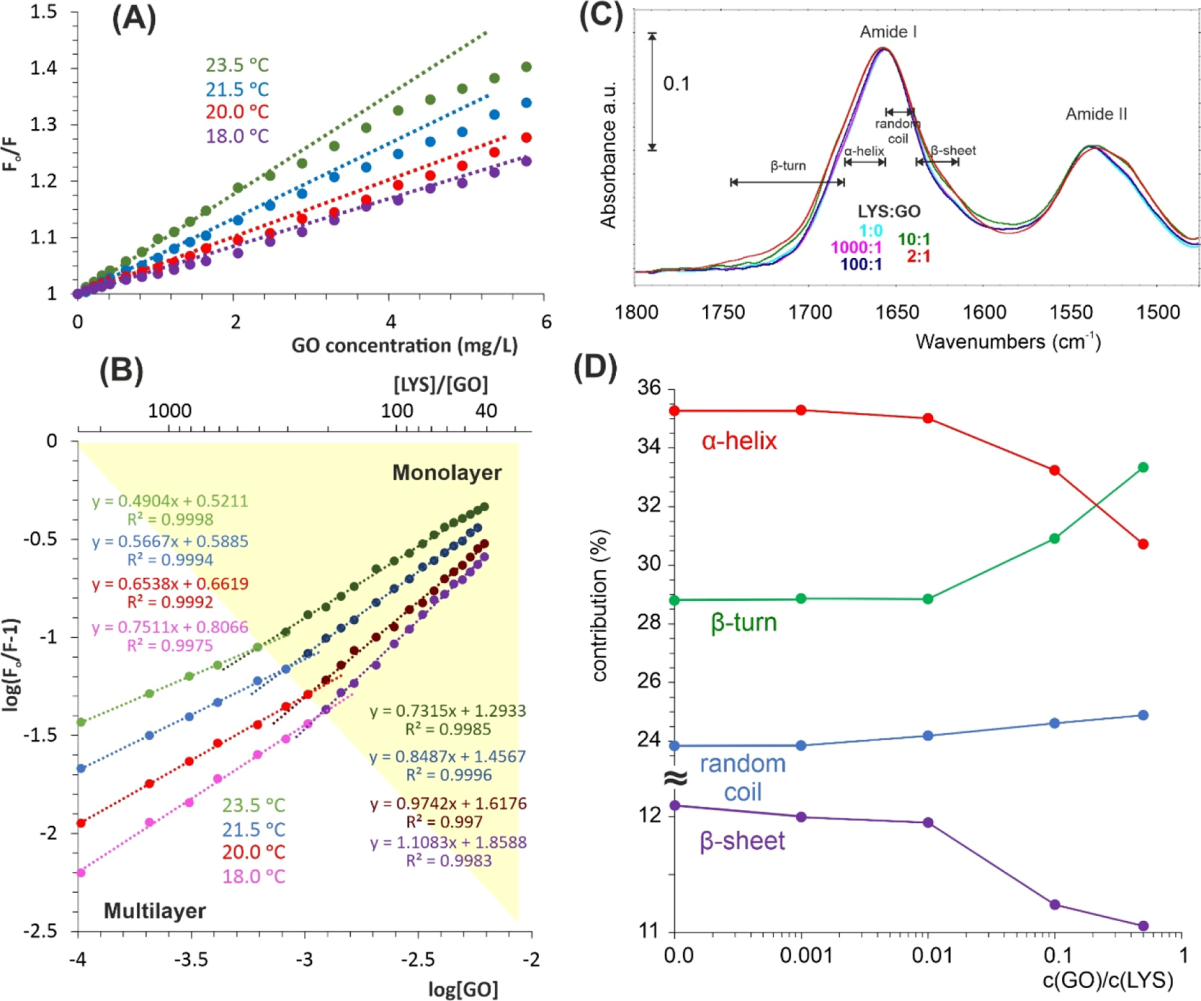
(A) Stern–Volmer and (B) log[(*F*_0_ – *F*)/*F*] vs log[GO] plots at different temperatures. The protein (250 mg/L) was excited at 295 nm. The labeled yellow region corresponds to the monolayer formation during LYZ adsorption. (C) Secondary structure spectral changes as a result of protein adsorption on GO. Quantitative results (D) were obtained based on deconvolution, as shown in Figure S3.

Nevertheless, for all tested systems, positive cooperativity is observed, which means that no steric hindrance (observed, e.g., for catalase adsorption on GO^7^) takes place (see also the results presented in Figures 2C and S5). Moreover, the value of *n*_*x*_ below unity appears here due to nonspecific interaction, typical for physical adsorption. As a small and rigid molecule, LYZ has a high degree of freedom in movement, both rotational and lateral (see further results).

To prove that LYS is a good adsorbate, the results from additional experiments need to be examined: (i) thermodynamic parameters calculated from the binding constants (*K*_b_) based on fluorescence spectrometry and (ii) secondary structure changes from FTIR measurements.

The aromatic, protein fluorophores–tryptophan, tyrosine, and phenylalanine (where tryptophan contributes maximally to the fluorescence)^17^–are very sensitive to their microenvironment and thus useful for studying the protein binding associated with conformational changes. To investigate the mechanism and thermodynamics of GO–LYZ interactions, the fluorescence quenching data at 23.5, 21.5, 20.0, and 18.0 °C were analyzed by the LYZ fluorescence intensity decrease after the gradual increase of GO concentration, as shown in Figure 2B.

Due to the lack of linearity and possibility of GO molar weight determination, the values of *K*_sv_ and *k*_q_ - usually obtained from the Stern–Volmer (S–V) relation (Figure 2A) – cannot be established.

Nevertheless, the physicochemical characteristic of the quencher used (GO), the results presented in Figure 1, and our conclusions from previous studies^7^ allowed us to assume that the quenching process was not initiated by dynamic diffusion but by the formation of a complex between LYZ and GO. Further, the negative deviations from linearity on the S–V plot mean that more than one active site is needed at the low GO concentration region (multilayer adsorption dominates). The number of active sites increases with the temperature decrease and GO concentration increase, confirming the physical characteristic of LYZ adsorption on GO.

Additionally, FTIR analysis was performed to prove that physical adsorption occurs in the tested region of [LYZ]/[GO]. FTIR spectroscopy was applied to monitor the possible conformational changes of LYZ. It is well known that the analysis of the amide I band at approximately 1750–1600 cm^-1^ gives satisfactory information on the possible changes in the protein secondary structure.^7,18^ This band consists of several overlapping components that are recognized already after the analysis of the second derivative or self-deconvolution (Figure S4). The bands are assigned to different secondary structure elements (α-helix, β-sheets, β-turns, and random coil).^19,20^ The spectral decomposition (Figure S5) of the native and immobilized enzyme on GO shows only minor changes in the secondary structures, as shown in Figure 2C,D. From these data, it is obvious that very slight structural changes occur in the adsorption region. While the contribution of α-helices and β-turns remains unchanged, a slight increase in random coil at the expense of β-sheet can be observed.

Also, the increase in the [GO]/[LYZ] ratio up to 0.1 causes significant changes in the secondary structure. It is known from the literature^7,21^ that β-turns are considered as the final structures formed by the reorganization of some amino acid residues.

Therefore, the described results stay in good agreement with the ones from the adsorption data and mean that due to nonspecific adsorption the protein molecules have a high degree of freedom in movement, which can lead to a gentle reorganization of the secondary structure and to an increase of enzymatic activity.

While the quenching mechanism concerns the adsorptive complex formation, the binding constant, *K*_b_, can be calculated according to eq S9 from the *y*-axis intercept of the plot of log[(*F*_0_ – *F*)/*F*] versus log[GO] (Figure 2B). It can be seen that two regions can be detected at each temperature, and the transition perfectly matches with the [LYZ]/[GO] ratio, where the protein monolayer is formed on the GO surface (Figure 1).

**Figure 3:**
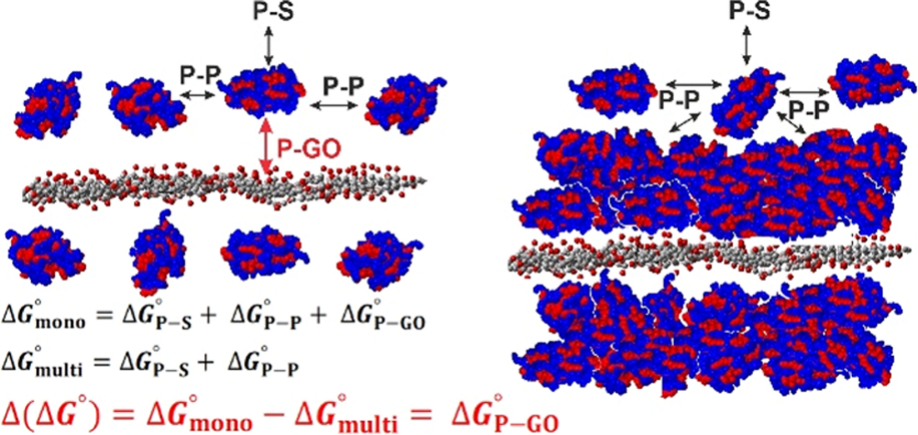
Schematic representation of interactions in the tested system: P–protein; S–solvent, and GO–graphene oxide.

The values of *K*_b,*x*_ (*x* = 1,2), for high and low [LYZ]/[GO] ratios, reflect multi- and monolayer formation. Thus, values obtained at different temperatures are listed in Figure 2B. While we cannot draw conclusions from absolute values (as we do not know the quencher’s molar mass), the *K*_2_/*K*_1_ ratio according to Figure 3 and eqs S12 and S13 represent directly the GO–LYZ interactions observed during the monolayer formation process. While in the multilayer region only LYZ–solvent and LYZ–LYZ interactions dominate, the increase in GO concentration—up to the monolayer region formation–causes an additional LYZ–GO energy appearance.

Based on the above findings, Δ(Δ*G*^0^), Δ(Δ*H*^0^), and Δ(Δ*S*^0^) for the interactions between LYZ and GO can be calculated according to thermodynamic relations (eq S10) and the van’t Hoff equation (eq S11). The values obtained are listed in Table 2.

**Table 2:** . Relative Thermodynamic Parameters in the GO–LYZ System^a^

temp. °C	*K*_b,2_/*K*_b,1_	Δ(Δ*G*^0^) kJ/mol	Δ(Δ*H*^0^) kJ/mol	Δ(Δ*S*^0^) J/mol K
23.5	5.92	–4.39		
21.5	7.38	–4.90		
20.0	9.03	–5.36	–195.0	–621.3
18.0	11.28	–5.86		
4.0	66.00	–9.65		

a The shadowed row concerns lysozyme adsorption on GO at 4 °C, and values of *K*_2_ and *K*_1_ were obtained from the L–F model (eq 1) (see Figure S4).

Formally, the signs and magnitude of thermodynamic parameters [Δ(Δ*H*^0^) and Δ(Δ*S*^0^)] can be harnessed to determine the main energies that contribute to adsorptive complex formation.^22^ The exothermic nature and dominant involvement of electrostatic interactions can be concluded from the negative Δ(Δ*H*^0^) value. Nevertheless, the hydrogen bonds, according to Ross and Subramanian,^22^ also play a significant role, as depicted from the negative values of both of Δ(Δ*H*^0^) and Δ(Δ*S*^0^). Furthermore, the slightly negative sign of Δ(Δ*S*^0^) confirms the physical adsorption character of LYZ–GO interactions due to the hydrogen-bond formation. Thus, one can conclude that the binding process is enthalpy-driven, as Δ(Δ*H*^0^) contributes more to Δ(Δ*G*^0^) than Δ(Δ*S*^0^). In addition, the negative sign of Δ(Δ*G*^0^) confirms that the adsorption process is spontaneous.

The description of LYZ adsorption isotherms on GO with the bimodal Langmuir–Freundlich (eq 1) results in the appearance of two constants describing mono- and multilayer formation (Table 1). As the theory is correct, the *K*_2_/*K*_1_ ratio should be fitted in the thermodynamic parameter changes. Figures S3 and S4 confirms this assumption, showing that the simple theory is very useful for the determination of GO SSA.

## 4 Conclusions

Concluding, we prove that (i) LYZ is the perfect adsorbate for GO SSA determination; (ii) the bimodal Langmuir–Freundlich model allows for the trustworthy description of the adsorption phenomena; (iii) the thermodynamic description of the adsorption process is possible, even for unknown molar mass adsorbents; and (iv) the LYZ–GO interactions are mainly electrostatic in nature, confirming physical adsorption.

## Supporting Information Available

Summary of the literature data on adsorption capacity with the use of MB as the adsorbate; MB and LYZ adsorption isotherms with fitted elements; K2/K1 ratio as a function of ultrasonication time; and Δ(ΔG°) temperature dependence leading to Δ(ΔH°) and Δ(ΔS°) determination (PDF)
