# Supplementary material for: Determination of Graphene Oxide Adsorption Space by Lysozyme Uptake—Mechanistic Studies
Source: J Phys Chem B. 2022 Jan 25;126(4):928–33. doi: 10.1021/acs.jpcb.1c08294 (PMC8819649; doi:10.1021/acs.jpcb.1c08294)
Supplement: Supplementary file 1 — jp1c08294_si_001.pdf [file jp1c08294_si_001.pdf]

Supporting Information

Determination of Graphene Oxide Adsorption Space by Lysozyme Uptake – Mechanistic Studies

Paulina Erwardt [†,*], Katarzyna Roszek [‡], Marek Wiśniewski[†,*]

† Faculty of Chemistry, Physicochemistry of Carbon Materials Research Group, Nicolaus Copernicus University in Toruń, Gagarina 7, 87-100 Toruń, Poland

‡ Department of Biochemistry, Faculty of Biological and Veterinary Sciences, Nicolaus Copernicus University in Toruń, Lwowska 1, 87 – 100 Toruń, Poland

(*) corresponding authors

Email: marekw@umk.pl (Marek Wiśniewski)

Email: pbolibok@umk.pl (Paulina Erwardt)

Table S1. Summary of literature data on adsorption capacity with the use of methylene blue as adsorbate.

**Time of sonication [min]**	**T [K]**	**Q_m [mg/g]**	**SSA_{MB} [m^2/g]**	**Ref.**
no data	293	241	590	(1)
no data	RT	90	221	(2)
no data	298	351	860	(3)
no data	no data	301	737	(4)
360 during the adsorption process	no data	318	780	(5)
no data	no data	17	42	(6)
no data	298	357	875	(7)
no data	298	380	931	(8)
without	RT	505	1237	(9)
120	303	322	788	(10)
15	298	599	1467	(11)
no data	293	476	1167	(12)
15	298	515	1259	(13)
5	298	325	795	This study
10		436	1069	
15		448	1099	
20		424	1039	

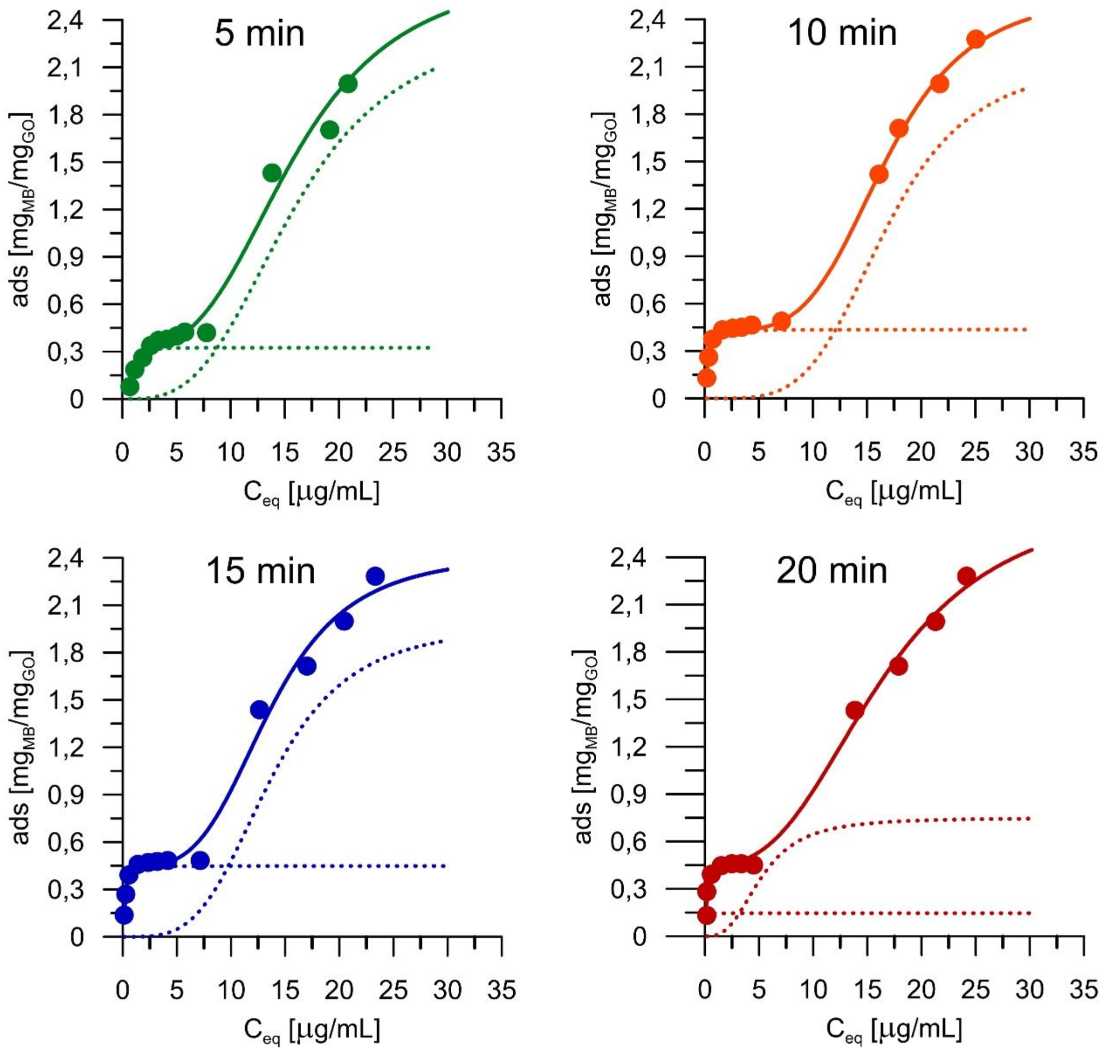

Figure S1. MB adsorption isotherms with fitted elements.

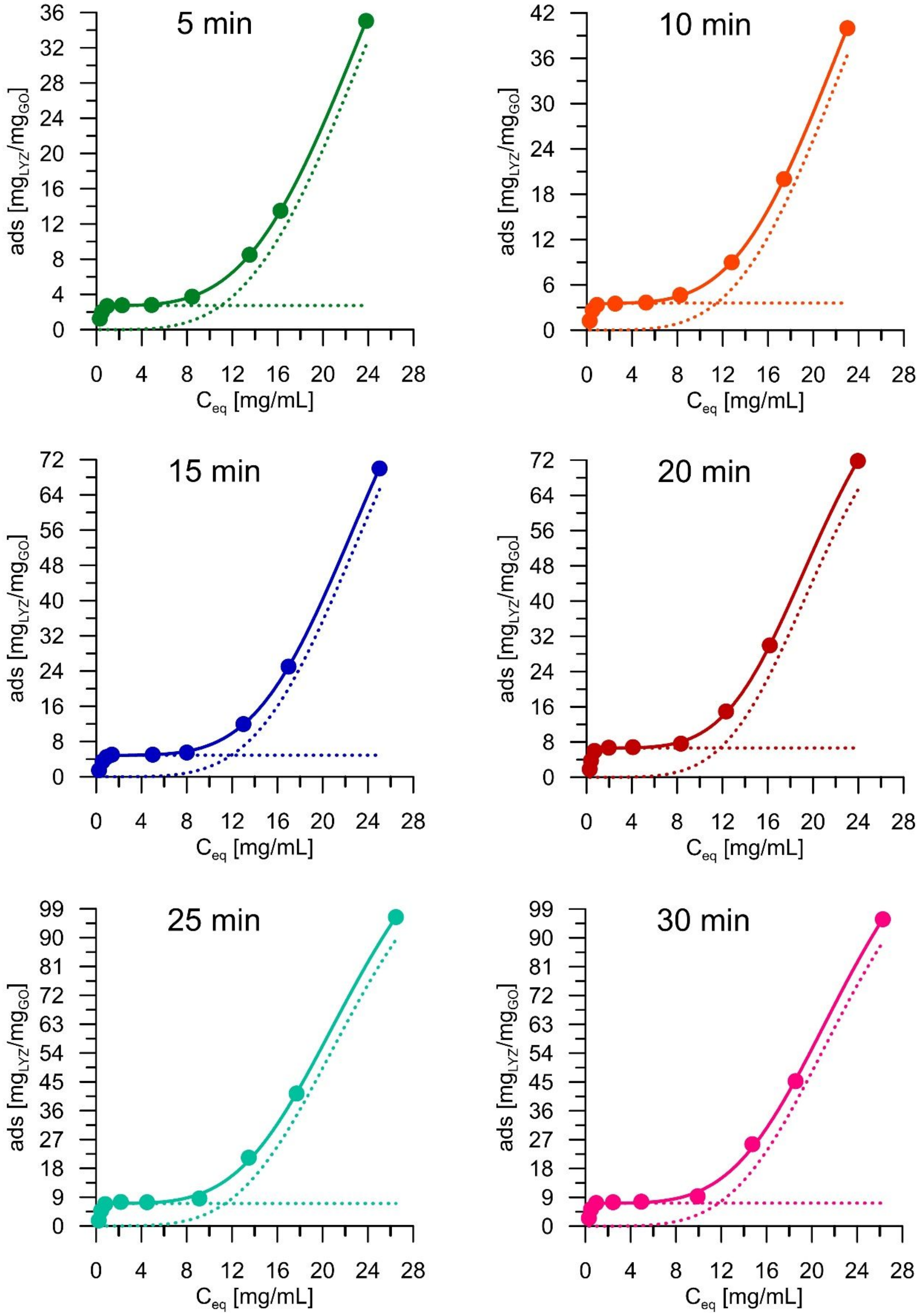

Figure S2. LYZ adsorption isotherms with fitted elements.

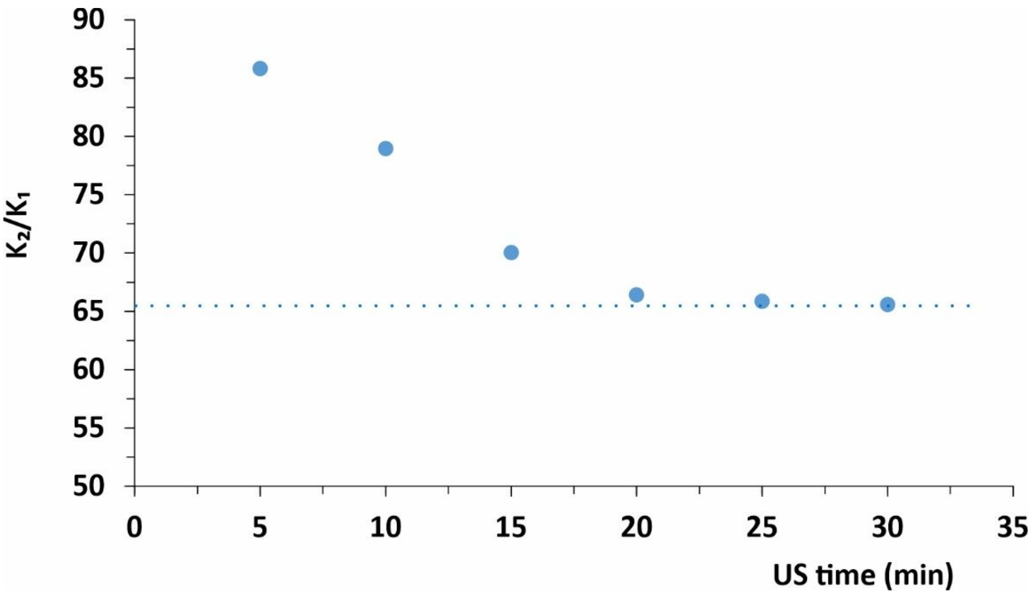

Figure S3. K_2/K_1 (from bimodal L-F model Eq. 1) ratio as a function of ultrasonication time.

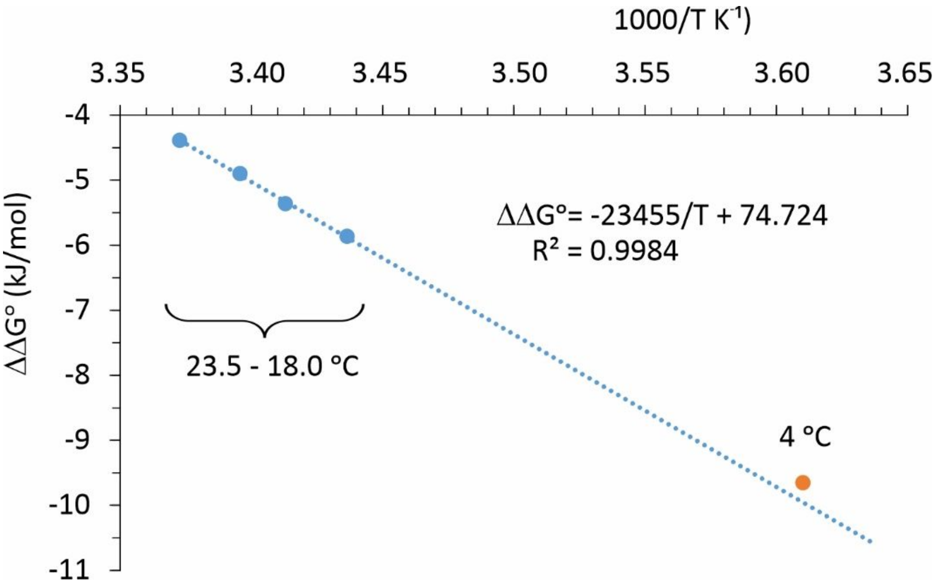

Figure S4. Δ(ΔG°) temperature dependence leading to Δ(ΔH°) and Δ(ΔS°) determination.

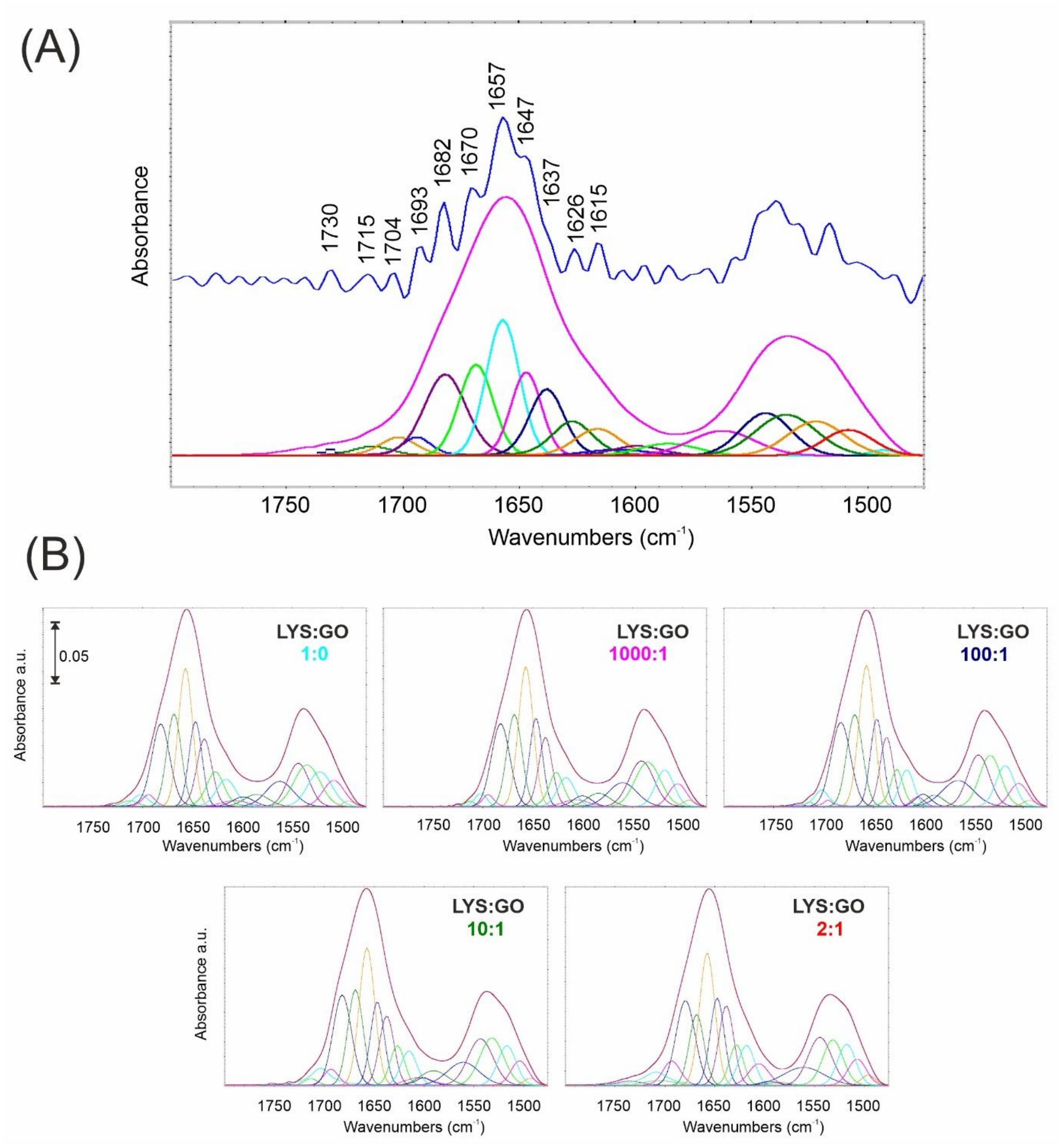

Figure S5. A – the amide I &II region FTIR spectrum of native LYZ with self-deconvolution; B – the spectral decomposition of amide I & II bands of native and adsorbed protein.

When simple relation

$$Q + B \rightleftarrows QB \tag{S1}$$

occurs, the fluorescence data were analyzed according to the Stern–Volmer equation to obtain Stern–Volmer quenching (K_{sv});

$$\frac{F_o}{F} = K_{SV}[Q] + 1 \tag{S2}$$

where, F_o and F are the fluorescence intensities in the absence and presence of quencher (GO).

If more complicated complex, Q_nB, is formed with protein B and have "n" multiple (equivalent and independent) binding sites then quenching reaction can be represented as:

$$nQ + B \rightleftarrows Q_nB, \tag{S3}$$

its binding constant (K_b) is given by equation:

$$K_b = \frac{[Q_nB]}{[Q]^n[B]}. \tag{S4}$$

Assuming $[B_o]$ as initial concentration and $[B]$ being unbounded protein concentration

$$[Q_nB] = [B_o] - [B] \tag{S5}$$

and

$$K_b = \frac{[B_o] - [B]}{[Q]^n[B]}. \tag{S6}$$

It is well known that the fluorescence intensity is proportional to the protein concentration, thus:

$$\frac{F_o}{F} = \frac{[B_o]}{[B]}. \tag{S7}$$

Then, relationship between the fluorescence intensity changes and unbound protein is expressed as:

$$K_b[Q]^n = \frac{[F_o]}{[F]} - 1, \tag{S8}$$

or in logarithmic form:

$$\log\left(\frac{F_o}{F} - 1\right) = \log\left(K_b\right) + n\log\left([Q]\right). \tag{S9}$$

On the other hand, the changes in standard Gibbs free energy (ΔG°) can be obtained using Gibbs-Helmholtz equation:

$$\Delta G^{\circ} = -RT\ln(K_b), \tag{S10}$$

while, change in standard enthalpy (ΔH°) and entropy (ΔS°) are usually determined from the linearization (K_b vs. T^{-1}) van't Hoff equation:

$$\ln\left(K_b\right) = -\frac{\Delta H^{\circ}}{RT} + \frac{\Delta S^{\circ}}{R}. \tag{S11}$$

The main problem here is fact that GO molar mass is unknown and cannot be designated. As consequence, thermodynamic parameters remain undefined.

By assuming the interactions energies in multilayer (LYZ-LYZ and LYZ-solvent) and in monolayer (LYZ-LYZ, LYZ-solvent and LYZ-GO), the difference $\Delta(\Delta G^{\circ})$ describes pure interaction between GO and LYZ (see Fig.3):

$$\Delta G^{\circ}_{LYZ-GO} = \Delta(\Delta G^{\circ}) = \Delta G^{\circ}_{mono} - \Delta G^{\circ}_{multi} = -RT\ln\left(\frac{K_{b,2}}{K_{b,1}}\right). \tag{S12}$$

In consequence, $\Delta H^{\circ}_{LYZ-GO}$ and $\Delta S^{\circ}_{LYZ-GO}$ can also be determined:

$$\ln\left(\frac{K_{b,2}}{K_{b,1}}\right) = -\frac{(\Delta H_2^\circ - \Delta H_1^\circ)}{RT} + \frac{(\Delta S_2^\circ - \Delta S_1^\circ)}{R} = -\frac{\Delta(\Delta H^\circ)}{RT} + \frac{\Delta(\Delta S^\circ)}{R}, \quad \text{(S13)}$$

where indexes 1 and 2 are respectively for multi- and monolayer of LYZ on GO.

Literature:

(1) Li, Y.; Du, Q.; Liu, T.; Peng, X.; Wang, J.; Sun, J.; Wang, Y.; Wu, S.; Wang, Z.; Xia, Y.; Xia, L. Comparative study of methylene blue dye adsorption onto activated carbon, graphene oxide, and carbon nanotubes. *Chem. Eng. Res. Des*. **2013**, *91*(2), 361–368.
(2) Sahoo, S. K.; Purohit, A. K.; Panigrahi, G. K.; Sahoo, J. K. Adsorption of methylene blue from aqueous media using graphene oxide Synthesised from bio-soot wastes. *Phys. Chem. Liq.* **2020**, 1-11.
(3) Bradder, P.; Ling, S. K.; Wang, S.; Liu, S. Dye adsorption on layered graphite oxide. *J. Chem. Eng. Data.* **2011**, *56*(1), 138–141.
(4) Montes-Navajas, P.; Asenjo, N. G.; Santamaría, R.; Menéndez, R.; Corma, A.; García, H. Surface Area Measurement of Graphene Oxide in Aqueous Solutions. *Langmuir* **2013**, *29* (44), 13443–13448.
(5) Chen, L.; Batchelor-McAuley, C.; Rasche, B.; Johnston, C.; Hindle, N.; Compton, R. G. Surface area measurements of graphene and graphene oxide samples: Dopamine adsorption as a complement or alternative to methylene blue?. *Appl. Mater. Today* **2020**, *18*, 100506.
(6) Ramesha, G. K.; Vijaya Kumara, A.; Muralidhara, H. B.; Sampath, S. Graphene and graphene oxide as effective adsorbents toward anionic and cationic dyes. *J. Colloid Interface Sci.* **2011**, *361*(1), 270–277.
(7) Abd-Elhamid, A. I.; Aly, H. F.; Soliman, H. A. M.; El-Shanshory, A. A. Graphene oxide: Follow the oxidation mechanism and its application in water treatment. *J. Mol. Liq.* **2018**, *265*, 226–237.
(8) Ding, S.; Sun, S.; Xu, H.; Yang, B.; Liu, Y.; Wang, H.; Chen, D.; Zhang, R. Preparation and adsorption property of graphene oxide by using waste graphite from diamond synthesis industry. *Mater. Chem. Phys.* **2019**, *221*, 47–57.
(9) de Araujo, C. M. B.; de Assis Filho, R. B.; Baptisttella, A. M. S.; do Nascimento, G. F. O.; da Costa, G. R. B.; Carvalho, M. N.; Ghislandi, M. G.; da Motta Sobrinho, M. A. Systematic study of graphene oxide production using factorial design techniques and its application to the adsorptive removal of methylene blue dye in aqueous medium. *Mater. Res. Express* **2018**, *5*(6), 065042.
(10) Lavin-Lopez, M. P.; Patón-Carrero, A.; Muñoz-Garcia, N.; Enguilo, V.; Valverde, J. L.; Romero, A. The influence of graphite particle size on the synthesis of graphene-based materials and their adsorption capacity. *Colloids Surf. A Physicochem. Eng. Asp.* **2019**, *582*, 123935.
(11) Yan, H.; Tao, X.; Yang, Z.; Li, K.; Yang, H.; Li, A.; Cheng, R. Effects of the oxidation degree of graphene oxide on the adsorption of methylene blue. *J. Hazard. Mater.* **2014**, *268*, 191–198.
(12) Chia, C.; Nur, F. R.; Mohd, S. S.; Sarani, Z.; Huang, N.; Lim, H. Methylene blue adsorption on graphene oxide. *Sains Malays.* **2013**, *42*(6), 819–826.
(13) Bolibok, P.; Koter, S.; Kaczmarek-Kędziera, A.; Kowalczyk, P.; Łukomska, B.; Łukomska, O.; Boncel, S.; Wiśniewski, M.; Kaneko, K.; Terzyk, A. P. Liquid phase adsorption induced nanosizing of graphene oxide. *Carbon* **2021**, *183*, 948–957.
